# Reducing HDAC6 ameliorates cognitive deficits in a mouse model for Alzheimer's disease

**DOI:** 10.1002/emmm.201201923

**Published:** 2012-11-26

**Authors:** Nambirajan Govindarajan, Pooja Rao, Susanne Burkhardt, Farahnaz Sananbenesi, Oliver M Schlüter, Frank Bradke, Jianrong Lu, André Fischer

**Affiliations:** 1 Department of Psychiatry and Psychotherapy, University Medical Center, Georg-August-University GoettingenGoettingen, Germany; 2 German Center for Neurodegenerative Diseases (DZNE)Goettingen, Germany; 3 Molecular Neurobiology, European Neuroscience Institute GoettingenGoettingen, Germany; 4 German Center for Neurodegenerative Diseases (DZNE) BonnBonn, Germany; 5 Department of Biochemistry and Molecular Biology, University of FloridaGainseville, FL, USA

**Keywords:** Alzheimer's disease, cognition, epigenetics, histone deacetylase, neurodegeneration

## Abstract

Histone deacetylases (HDACs) are currently being discussed as promising therapeutic targets to treat neurodegenerative diseases. However, the role of specific HDACs in cognition and neurodegeneration remains poorly understood. Here, we investigate the function of HDAC6, a class II member of the HDAC superfamily, in the adult mouse brain. We report that mice lacking HDAC6 are cognitively normal but reducing endogenous HDAC6 levels restores learning and memory and α-tubulin acetylation in a mouse model for Alzheimer's disease (AD). Our data suggest that this therapeutic effect is, at least in part, linked to the observation that loss of HDAC6 renders neurons resistant to amyloid-β-mediated impairment of mitochondrial trafficking. Thus, our study suggests that targeting HDAC6 could be a suitable strategy to ameliorate cognitive decline observed in AD.

## INTRODUCTION

Alzheimer's disease (AD) is an age-associated neurodegenerative disorder that causes severe impairment of cognitive function leading to a drastic decline in the quality of life. Pathological features of AD are extracellular plaques, comprised of aggregated amyloid-β (Aβ) peptides, and intraneuronal tangles that consist of aggregated and hyperphosphorylated tau protein (Haass & Selkoe, 2007; Schneider & Mandelkow, 2008). These hallmarks are accompanied by loss of neurons, impairment of neuronal functions and brain atrophy (Karow et al, 2010; Nelson, 2005; Wenk, 2003). A minority of AD cases, referred to as familial AD, is caused by mutations in the amyloid precursor protein (APP) gene or genes that affect amyloid processing (Bertram & Tanzi, 2008). The vast majority of AD cases is however sporadic and characterized by late onset (Marques et al, 2010). Although there is now convincing data suggesting that Aβ peptides and soluble tau protein impair synaptic function, the precise cause of sporadic AD is not entirely understood (Ittner et al, 2010; Shankar et al, 2008) and effective therapies still await to be developed.

Recent research suggests that dysregulation of epigenetic processes, such as histone acetylation, might be causally linked to the pathogenesis of sporadic AD. Histone acetylation is regulated by the counteracting activities of histone acetyltransferases (HATs) and histone deacetylases (HDACs) and has been identified as a key process to regulate chromatin plasticity during memory formation (Levenson & Sweatt, 2005). Changes in histone acetylation seem to play a crucial role in the dysregulation of gene expression observed during AD and a number of studies have now repeatedly shown that targeting histone acetylation via administration of HDAC inhibitors can ameliorate cognitive deficits in AD animal models (Francis et al, 2009; Govindarajan et al, 2011; Kilgore et al, 2010; Peleg et al, 2010; Ricobaraza et al, 2009, 2012). Thus, HDAC inhibition is considered to be a novel promising therapeutic strategy to treat AD (Abel & Zukin, 2008; Fischer et al, 2010; Sananbenesi & Fischer, 2009).

The mammalian genome encodes 11 zinc-dependent HDAC proteins that are grouped into three classes. Class I HDACs are mainly localized to the nucleus and consist of HDAC1, HDAC2, HDAC3, and HDAC8. Class II HDACs are subdivided into class IIa (HDAC4, HDAC5, HDAC7, and HDAC9) and class IIb (HDAC6 and HDAC10). Class II HDACs can shuttle between the cytoplasm and nucleus. HDAC11 is the sole member of class IV (Gregoretti et al, 2004).

So far pan-HDAC inhibitors with limited specificity to individual HDAC proteins have been used in AD models. However, deeper insights into the role of distinct HDACs in brain function will be essential for further drug development. In addition, it is important to note that although the therapeutic effect of HDAC inhibition has been mainly discussed in the context of histone acetylation and gene expression, HDACs evolved in the absence of histone proteins and are known to target proteins other than histones in all subcellular compartments (Fischer et al, 2010). Thus, an important goal for future research is to gain a better understanding of the therapeutic effect of HDAC inhibition via histone as well as non-histone targets.

Here, we start to address these questions by studying the role of HDAC6 in memory function and AD pathogenesis. HDAC6 is a unique member of the HDAC family that acts mainly on cytoplasmic non-histone substrates (Haggarty et al, 2003; Valenzuela-Fernández et al, 2008; Zhang et al, 2006). A major HDAC6 substrate is α-tubulin acetylated at lysine 40 (α-tubulin K40ac) and in line with this observation, HDAC6 has been implicated in the regulation of cytoskeletal stability, intracellular transport and cell motility (Hubbert et al, 2002; Valenzuela-Fernández et al, 2008). By generating and analyzing *Hdac6* knockout mice (*Hdac6*^−/−^ mice), we show that loss of HDAC6 function increases hippocampal α-tubulin K40ac levels but has no effect on histone acetylation. *Hdac6*^−/−^ mice are viable and show no detectable cognitive dysfunction. However, when crossed with a model for severe amyloid pathology, reduction of HDAC6 levels ameliorated the impairment of α-tubulin K40ac and associative and spatial memory formation. Furthermore, we identify one cellular substrate to explain, at least in part, the therapeutic effect of reducing HDAC6 levels by showing that loss of *Hdac6* rescues Aβ-induced deficits in mitochondrial trafficking. In conclusion, our data suggest that HDAC6 might be a suitable molecular target for the development of novel therapeutic strategies against AD.

## RESULTS

### HDAC6 in the brain

**Figure 1:**
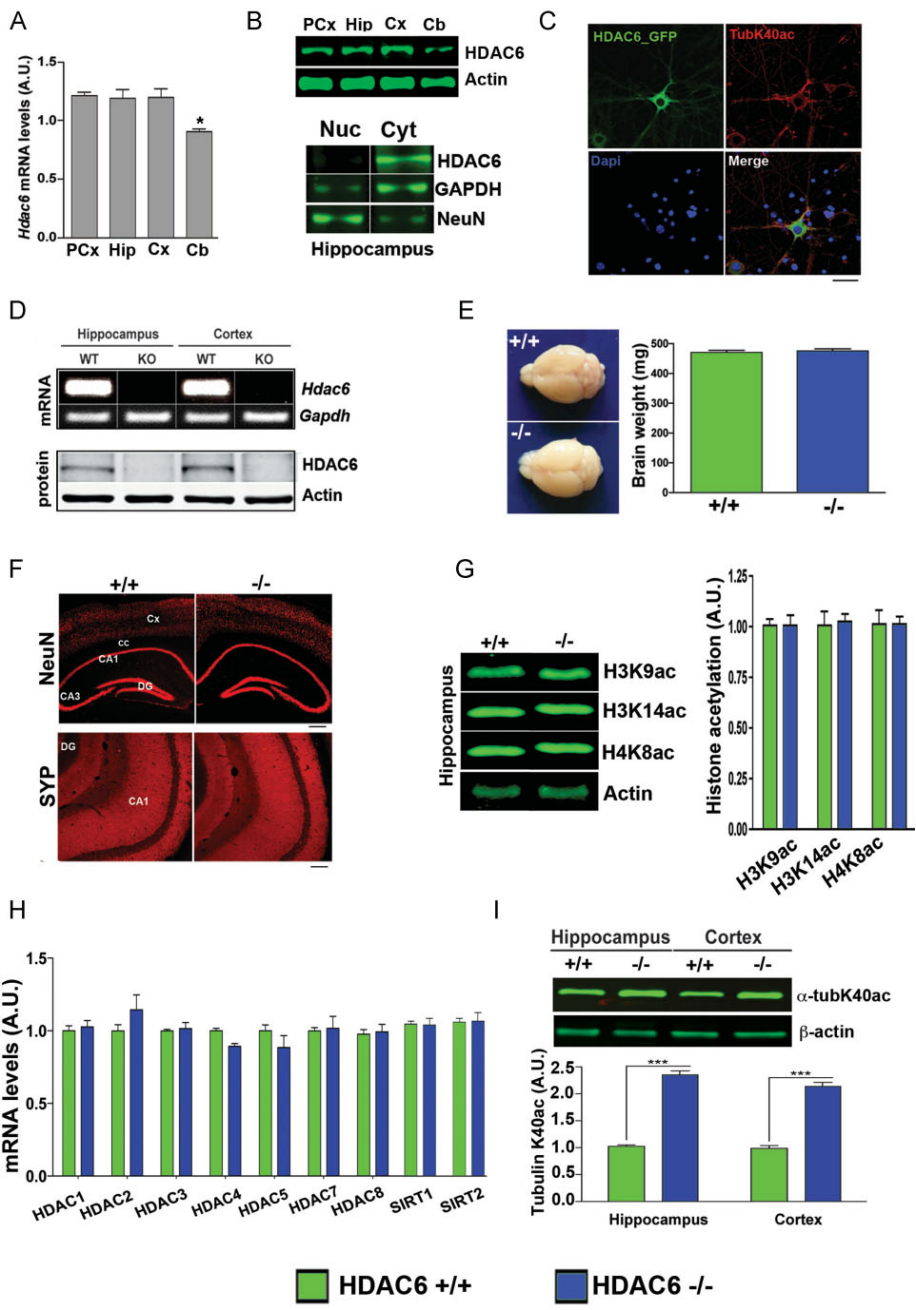
Loss of *Hdac6* increases α-tubulin acetylation in the hippocampus and cortex Error bars indicate SEM (*n* = 3, Student's *t*-test, *p* < 0.0001). PCx, pre-frontal cortex; Hip, hippocampus; Cx, cortex; Cb, cerebellum; Nuc, nuclear lysates; Cyt, cytoplasmic lysates; HDAC6^+/+^, wild type; HDAC6*^−/−^*, *Hdac6* knockout. Quantitative real-time PCR showing normalized *Hdac6* expression in different mouse brain regions. (*n* = 4, Student's *t*-test, PCx *versus* Cb: *p* = 0.0001, Hip *versus* Cb: *p* = 0.0107, Cx *versus* Cb: *p* = 0.0066).Upper panel: Immunoblot analysis showing the HDAC6 protein levels in different brain regions. Lower panel: Immunoblot showing the predominant localization of HDAC6 to the cytoplasm.Representative images showing cytoplasmic localization of viral-expressed HDAC6-GFP protein in primary hippocampal neurons (Scale bar: 10 µm).PCR (upper panel) and immunoblot analysis (lower panel) confirming loss of *Hdac6* mRNA and protein in the hippocampus and cortex of *Hdac6*^*−/−*^ mice.Representative brain images and brain mass in adult *Hdac6*^*−/−*^ and wild type mice.Representative images showing similar immunoreactivity of NeuN (scale bar: 100 µm) and SYP (scale bar: 50 µm) in *Hdac6*^*−/−*^ and wild type mice.Immunoblot (left) showing hippocampal histone acetylation in *Hdac6*^*−/−*^ mice and wild type littermates along with densitometric quantification (right).qPCR analysis of mRNA levels of other HDACs in *Hdac6*^*−/−*^ mice and wild type littermates.Quantitative immunoblot analysis showing elevated α-tubulin K40ac levels in the hippocampus and cortex of *Hdac6*^*−/−*^ mice compared to wild type mice.

We started our analysis by measuring HDAC6 levels in different regions of the adult mouse brain. Comparable levels of *Hdac6* mRNA were detected in the hippocampus and cortical regions, while a significantly lower expression of *Hdac6* was observed in the cerebellum (Fig 1A). This mRNA expression profile correlated with HDAC6 protein levels that were analysed via quantitative immunoblotting. HDAC6 protein levels were similar in the hippocampus and cortical regions but significantly lower in the cerebellum (Fig 1B, upper panel). Next, we analysed the subcellular localization of HDAC6 in the mouse hippocampus, a brain region important for the consolidation of memories and one of the first regions to be affected in AD patients (Mesulam, 1999). HDAC6 protein levels were mainly restricted to the cytoplasm and below detection level in the nucleus (Fig 1B, lower panel). This subcellular distribution of HDAC6 was confirmed in hippocampal neurons *in vitro*. Since we were unable to obtain an anti-HDAC6 antibody suitable for immunocytochemistry, we generated a lentivirus to express *Hdac6*-eGFP in primary hippocampal neurons. The *Hdac6*-eGFP signal was restricted to the cytoplasm (Fig 1C). To further analyse the role of HDAC6, we generated *Hdac6* knockout mice by replacing exons 10–13 of the mouse *Hdac6* gene with a neomycin cassette (*Hdac6*^*−/−*^ mice). This led to the complete loss of *Hdac6* mRNA and protein levels when measured in the hippocampus and cortex as shown by semi-quantitative PCR (Fig 1D, upper panel) and immunoblot, respectively (Fig 1D, lower panel). Notably, gross brain morphology and brain mass (Fig 1E) and body mass (Supporting Information Fig S1) were normal in *Hdac6*^*−/−*^ mice.

In line with this finding, we observed no significant differences in immunoreactivity against Neuronal N (NeuN) and synaptophysin (SYP), two well-established markers of neuronal and synaptic integrity, between *Hdac6*^*−/−*^ mice and wild type littermates (Fig 1F). Hippocampal bulk histone acetylation was not altered in *Hdac6*^*−/−*^ mice (Fig 1G). Similarly, the mRNA levels of class I and II HDACs and Sirtuins were unaffected (Fig 1H) and no changes were observed in the expression of some genes previously shown to be involved in memory consolidation (Govindarajan et al, 2011; Peleg et al, 2010; Supporting Information Fig S1). Expectedly, *Hdac6*^*−/−*^ mice exhibited significantly elevated levels of α-tubulin K40ac in the hippocampus and cortex shown by immunoblot (Fig 1I).

**Figure 2:**
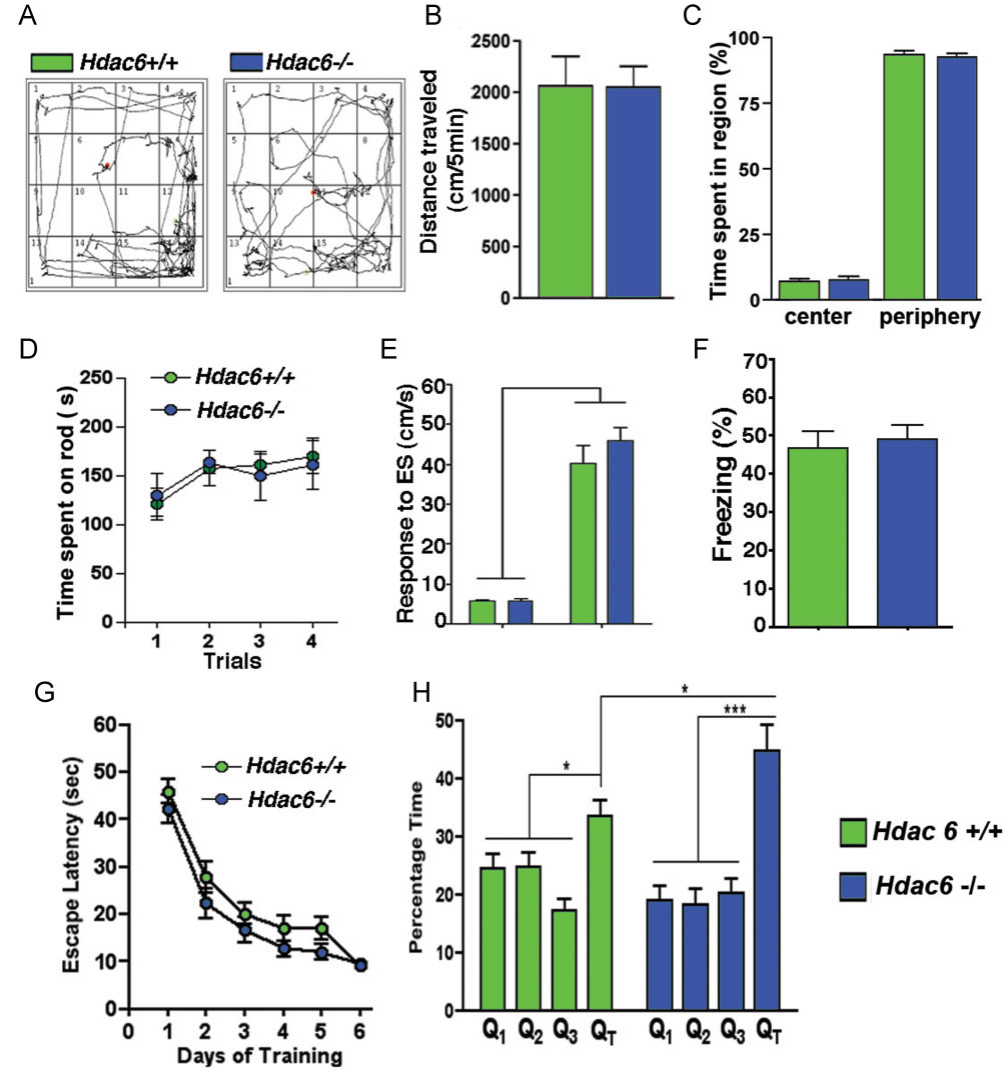
Behavioral analysis of *Hdac6* *^−/−^* mice Representative images showing exploratory behaviour in the open field test.Total distance covered during a 5 min open field exposure by *Hdac6*^*−/−*^ and wild type mice (*n* = 10).The time spent in the center *versus* periphery of the open field in *Hdac6*^*−/−*^ and wild type mice (*n* = 10).Motor function was analysed in the Rotarod test. Time spent on the rotating rod in *Hdac6*^*−/−*^ and wild type mice (*n* = 10).The total activity and the response to the electric foot shock in the contextual fear conditioning paradigm in *Hdac6*^*−/−*^ and wild type mice (*n* = 10).Contextual freezing behaviour assessed 24 h after the training in *Hdac6*^*−/−*^ and wild type mice (*n* = 10).Escape latency during the training phase of the Morris water maze in *Hdac6*^*−/−*^ and wild type mice (*n* = 15).Time spent in the target quadrant during the probe test in *Hdac6*^*−/−*^ and wild type mice (*n* = 15). Values are mean ± SEM *p = 0.0384, ***p = 0.0001, analysed by Student's *t*-test. *Q*_T_, Target quadrant.

Next, we subjected *Hdac6*^*−/−*^ and wild type mice to behavioural testing. Exploratory behaviour was unaltered in *Hdac6*^*−/−*^ mice when compared to wild type littermates. Both groups showed indistinguishable activity when exposed to a novel context (Fig 2A and B). Moreover, the time spent in the centre *versus* periphery of the open field was similar between groups indicating no changes in basal anxiety (Fig 2C). Motor coordination that was measured using the accelerating rotarod test was similar in *Hdac6*^*−/−*^ mice and wild type littermates (Fig 2D). When exposed to contextual fear conditioning, a robust test to assess associative memory function, *Hdac6*^*−/−*^ mice showed normal activity during the training and the response to the electric foot shock was indistinguishable from the control group (Fig 2E). Long-term associative memory, measured via the assessment of freezing behaviour upon re-exposure to the conditioning context 24 h after the training was not different between the two groups (Fig 2F). Finally, we measured spatial learning using the Morris Water Maze paradigm, a well-established and sensitive test to measure hippocampus-dependent spatial memory function. While the escape latency throughout 6 days of training was similar between groups (Fig 2G), during the subsequent memory test *Hdac6*^*−/−*^ mice showed a significantly increased preference for the target region when compared to the wild type control group (Fig 2H). Two-way ANOVA revealed no effect of gender on any of the above-described behaviour tests. In conclusion, these data suggest that loss of HDAC6 has no detrimental effect on brain morphology, motor coordination, or hippocampus-dependent cognitive function.

### Reducing *Hdac6* levels ameliorates AD cognitive pathology

**Figure 3:**
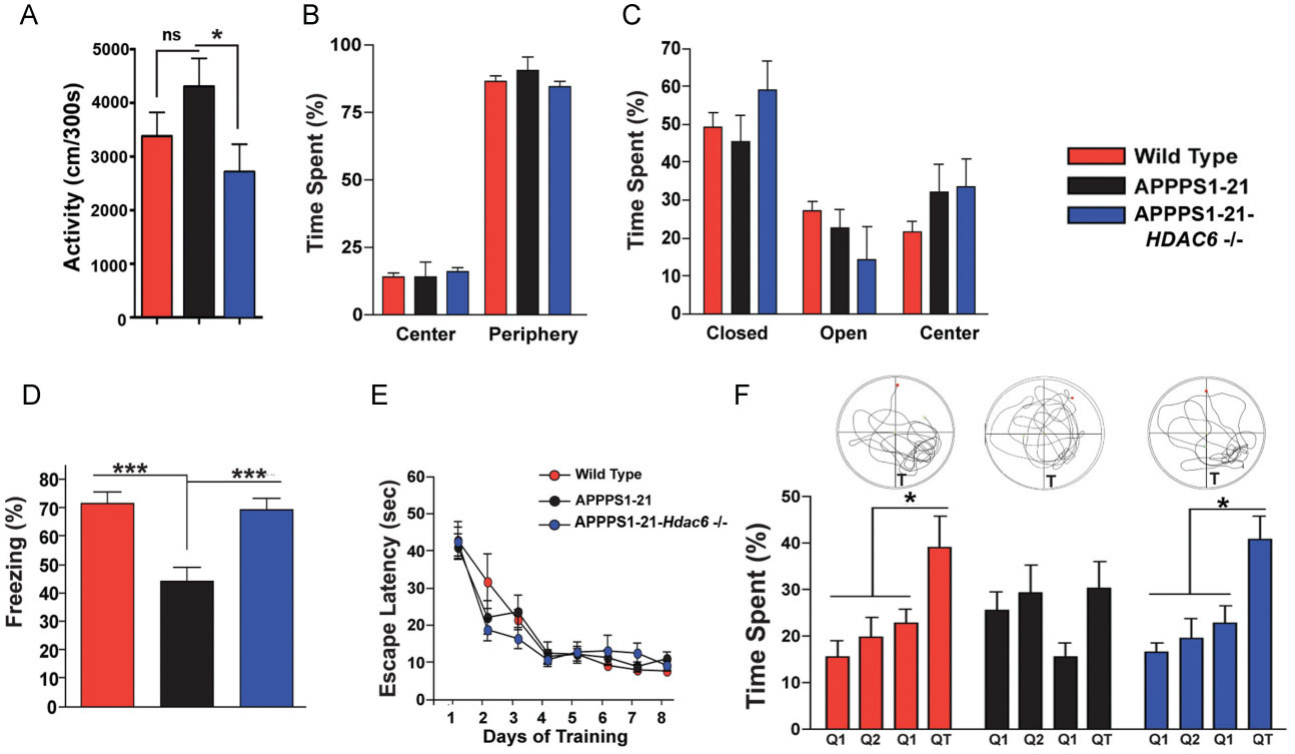
Loss of *Hdac6* rescues memory function in APPPS1-21 mice Total activity in the open field in wild type, APPPS1-21 and APPPS1-21-*Hdac6*^*−/−*^ mice (*n* = 8, Student's *t*-test, *p* = 0.0289).Time spent in the centre *versus* the periphery of the open field.Time spent in the centre and periphery of the elevated plus-maze in wild type, APPPS1-21 and APPPS1-21-*Hdac6*^*−/−*^ mice.Freezing behaviour analysed 24 h after fear conditioning training in wild type (*n* = 9), APPPS1-21 (*n* = 11) and APPPS1-21-*Hdac6*^*−/−*^ mice (*n* = 10), analysed by Student's *t*-test, *p* = 0.0005.Escape latency during the Morris water maze training in wild type, APPPS1-21 and APPPS1-21-*Hdac6*^*−/−*^ mice.Target preference analysed in the probe test by comparing times spent by wild type, APPPS1-21 and APPPS1-21-*Hdac6*^*−/−*^ mice in different quadrants on the water maze, analysed by Student's *t*-test, *p = 0.0036. The upper panel indicates representative swim paths during the probe test. Values are mean ± SEM.

While the functional consequences are unknown, recent data indicate that HDAC6 levels are dysregulated in postmortem brain samples from AD patients (Ding et al, 2008). To elucidate the role of HDAC6 in AD, *Hdac6*^*−/−*^ mice were crossed with APPPS1-21 mice (to yield APPPS1-21-*Hdac6*^*−/−*^ mice), which is a model for severe amyloid pathology that shows memory impairment at 8 months of age (Govindarajan et al, 2011; Radde et al, 2006). Thus, we decided to analyse cognitive function in 8-month-old APPPS1-21-*Hdac6*^*−/−*^ mice. APPPS1-21 and wild type littermates served as control groups. First, we tested exploratory behaviour in the open field paradigm. When compared to the wild type control group, the activities of APPPS1-21 or APPPS1-21-*Hdac6*^*−/−*^ mice were not significantly altered (Fig 3A). However, the direct comparison of APPPS1-21 to APPPS1-21-*Hdac6*^*−/−*^ mice revealed that loss of *Hdac6* rescued a mild hyperactivity phenotype observed in APPPS1-21 mice (Fig 3A). When the activity in the centre *versus* the periphery of the open field was analysed, no difference was observed between the groups, suggesting that basal anxiety levels were not altered (Fig 3B). In concordance with this observation, all groups showed similar performance in the elevated plus-maze test, another measure of basal anxiety (Fig 3C). In line with previous studies (Govindarajan et al, 2011), associative memory was impaired in the APPPS1-21 mice when compared to a wild type control group (Fig 3D). Interestingly, the impaired freezing behaviour exhibited by the APPPS1-21 mice was completely rescued to wild type levels in the APPPS1-21-*Hdac6*^*−/−*^ mice, which strongly suggests that loss of *Hdac6* restores associative memory function in APPPS1-21 mice (Fig 3D). Hippocampus-dependent memory function was also analysed in the Morris Water Maze test. All groups showed similar escape latencies during the 8 days of training (Fig 3E). When spatial memory function was assessed in the subsequent probe test, preference for the target quadrant was significantly impaired in APPPS1-21 mice when compared to wild type control group (Fig 3F). However, the comparison of APPPS1-21-*Hdac6*^*−/−*^ with APPPS1-21 mice revealed that target preference was significantly increased in APPPS1-21-*Hdac6*^*−/−*^ mice (Fig 3F). In conclusion, these data suggest that that loss of *Hdac6* can ameliorate associative and spatial memory impairment in a mouse model for AD.

**Figure 4:**
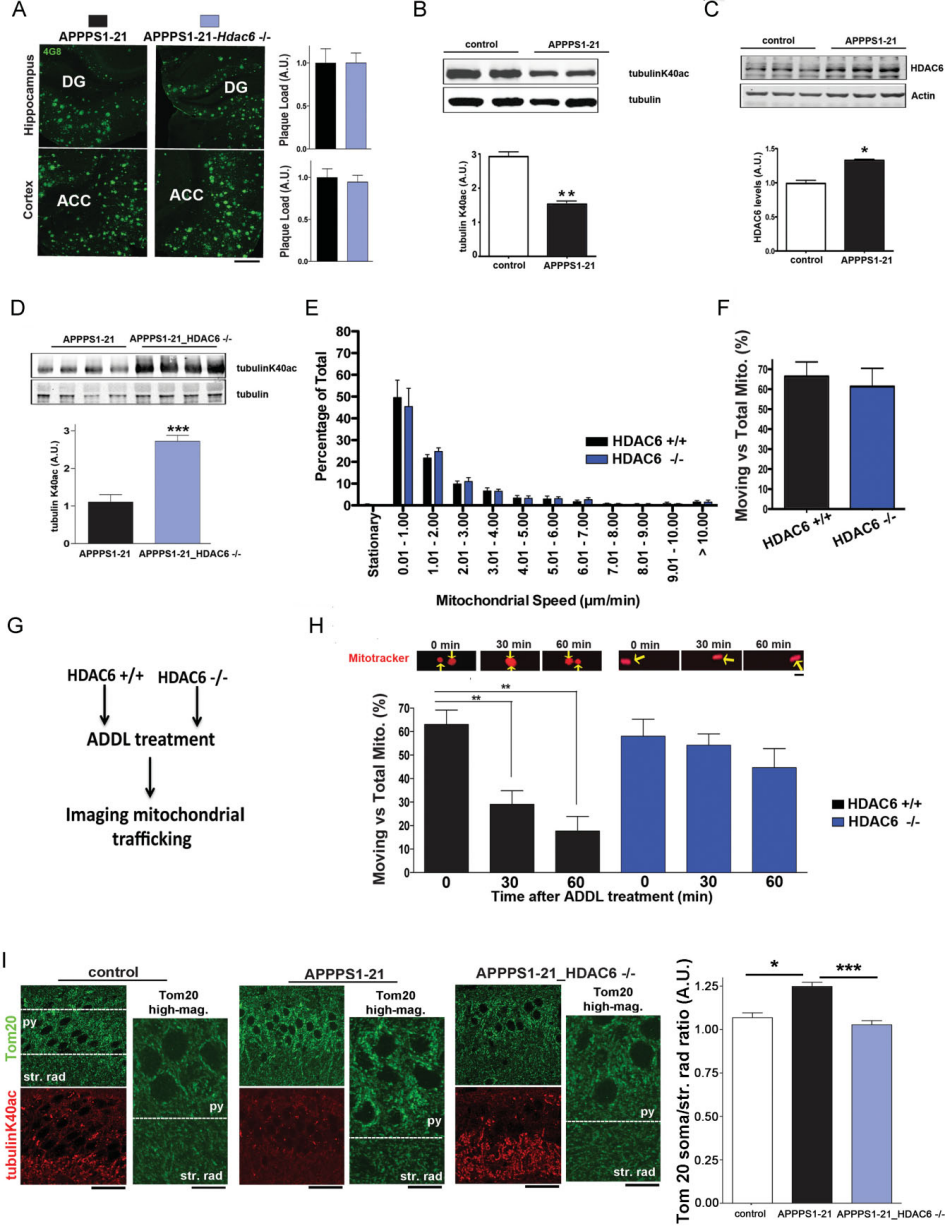
Loss of *Hdac6* rescues Aβ-induced impairment of mitochondrial trafficking ADDL, Aβ-derived diffusible ligand; py, hippocampal CA1 pyramidal cell layer; str. rad. Hippocampal stratum radiatum layer, DG, dentate gyrus; ACC, anterior cingulate cortex. Values are mean ± SEM. *p = 0.0112, ***p < 0.0001, analysed by Student's *t*-test. Left panel: Representative confocal microscopy images showing immunoreactivity against Aβ in the hippocampus and cortex of APPPS1-21 (*n* = 6) and APPPS1-21-*Hdac6*^*−/−*^ mice (*n* = 7). Scale bar: 100 µm. Right panel: Corresponding quantification of Aβ plaque load.Representative immunoblot (upper) and quantitative analysis (lower) showing reduced levels of α-tubulin K40 acetylation in APPPS1-21 mice compared to wild type animals (*n* = 6, Student's *t*-test, *p* = 0.0024).Representative immunoblot (upper) and quantitative analysis (lower) showing increased levels of HDAC6 in APPPS1-21 mice compared to wild type animals (*n* = 4, Student's *t*-test, *p* = 0.0212).Representative immunoblot (upper) and corresponding quantification (lower) showing increased levels of α-tubulin K40ac in the hippocampus of APPPS1-21-*Hdac6*^*−/−*^ compared to APPPS1-21 mice (*n* = 4, Student's *t*-test, *p* = 0.0007).Quantitative analysis depicting the distribution of mitochondrial trafficking at a distinct speed in primary hippocampal neurons isolated from *Hdac6*^*−/−*^ and wild type mice.Percentage of motile mitochondria out of total mitochondria in the experiment described under (**E**).Experimental design. Primary hippocampal neurons from *Hdac6*^*−/−*^ and wild type littermates were treated with ADDL for 30 or 60 min and mitochondrial trafficking was analysed.Upper panel: Representative time lapse images showing moving mitochondria in ADDL treated wild type and *Hdac6*^*−/−*^ neurons before (0 min) and 30 or 60 min after treatment. Scale bar: 5 µm. Lower panel: Quantitative analysis shows ADDL-mediated impairment in mitochondrial trafficking in wild type (0 min *vs.* 30 or 60 min) but not in *Hdac6*^*−/−*^ neurons (*n* = 6, Student's *t*-test, **p = 0.0053).Left panel: Representative images showing Tom20 and tubulinK40ac immunoreactivity in the hippocampus of wild type (*n* = 8), APPPS1-21 (*n* = 10) and APPPS1-21_HDAC6^*−/−*^ mice (*n* = 18). Dashed lines indicate areas used for quantification. Scale bar: 50 µm for low magnification images and 10 µm for Tom20 high magnification images (high-mag.). Right panel: Quantification of Tom20 immunoreactivity displayed as the ratio of intensity in soma to that in str. rad.

Preservation of associative and spatial memory function by reducing *Hdac6* is likely to involve multiple cellular processes. To better understand the mechanisms underlying the effect of HDAC6 on memory function in APPPS1-21 mice, we first analysed Aβ plaque load in APPPS1-21 and APPPS1-21-*Hdac6*^*−/−*^ mice. Immunohistochemical analysis revealed no difference between the two groups (Fig 4A). Taking into account that disturbances in cytoskeletal integrity play an important role during AD pathogenesis (Stokin et al, 2005), and that one of the best-described roles of HDAC6 is deacetylation of α-tubulin K40ac (Haggarty et al, 2003; Hubbert et al, 2002), we decided to analyze tubulin acetylation in APPPS1-21 and APPPS1-21-*Hdac6*^*−/−*^ mice.

First, we compared α-tubulin K40 acetylation in 10-month-old APPPS1-21 mice and an age-matched wild type control group. Interestingly, we found that hippocampal α-tubulin K40ac levels were significantly reduced in APPPS1-21 mice when compared to the wild type group (Fig 4B). In line with this observation, we detected a mild yet significant elevation of HDAC6 protein level in the hippocampi of APPPS1-21 mice compared to wild type controls (Fig 4C).

Interestingly, on comparing the levels of hippocampal α-tubulin K40ac in APPPS1-21 *versus* APPPS1-21-*Hdac6*^*−/−*^ mice, we observed that loss of *Hdac6* significantly increased α-tubulin K40 acetylation in APPPS1-21 mice (Fig 4D). This finding is of great importance since tubulin dynamics are known to be crucial for intracellular transport mechanisms, which are dysregulated in several neurodegenerative diseases including AD and in models of amyloid pathology (Butler et al, 2007; Hempen & Brion, 1996; Henriques et al, 2010). For example, impaired mitochondrial trafficking has been observed in models of amyloid pathology (Rui et al, 2006; Wang et al, 2010). Thus, we hypothesized that loss of HDAC6 may have an impact on dysregulated intracellular transport. To test this hypothesis, we analysed intraneuronal mitochondrial trafficking in primary hippocampal neurons from wild type and *Hdac6*^*−/−*^ mice in response to treatment with Aβ-derived diffusible ligands (ADDLs) using live cell imaging. The speed of mitochondrial trafficking (Fig 4E) and the percentage of moving mitochondria out of total mitochondria (Fig 4F) were not significantly altered when primary neurons derived from wild type and *Hdac6*^*−/−*^ mice were analysed. Consistent with previous findings (Rui et al, 2006), treatment of wild type hippocampal neurons with ADDLs resulted in a progressive impairment of mitochondrial trafficking (Fig 4H). In contrast, when hippocampal neurons obtained from *Hdac6*^*−/−*^ mice were treated with ADDLs, no such effect was observed (Fig 4H), suggesting that loss of *Hdac6* protects hippocampal neurons against Aβ-induced impairment in intracellular mitochondrial trafficking.

To investigate whether this result translates into the *in vivo* situation we analysed immunoreactivity to Tom20, a commonly used marker for mitochondrial localization (MacAskill et al, 2009), in wild type, APPPS1-21 and APPPS1-21-*Hdac6*^*−/−*^ mice. Accumulation of mitochondria at the somata of nerve cells has been described in the brain of AD patients and is interpreted as impaired intraneuronal mitochondrial trafficking contributing to disease pathogenesis (Wang et al, 2009). Thus, we quantified hippocampal immunoreactivity to Tom20 at the somata *versus* the stratum radiatum. We observed a significant increase in the ratio of Tom20 immunoreactivity in the soma to that in the stratum radiatum in APPPS1-21 mice when compared to the wild type control group (Fig 4I) indicative of impaired mitochondrial trafficking resulting in somatic accumulation of mitochondria in APPPS1-21 mice. Notably, this ratio in APPPS1-21-*Hdac6*^*−/−*^ mice was significantly lower than APPPS1-21 mice and comparable to wild type controls (Fig 4I) providing further evidence that the neuroprotective effect of loss of *Hdac6* is, at least in part, linked to improved mitochondrial trafficking.

## DISCUSSION

Since HDACs have emerged as potential therapeutic targets to treat neurodegenerative diseases, it is of utmost importance to study the role of individual HDACs in brain function. Here we have investigated the role of HDAC6 in memory function and the pathogenesis of AD. We show that *Hdac6* is expressed in regions of the adult brain that are implicated in memory function, namely hippocampus and cortex. Within the hippocampal formation HDAC6 was predominantly localized to the cytoplasm and absent from the nucleus, which is in line with previous reports on *Hdac6* expression in other cell types (Hubbert et al, 2002). To further study the role of HDAC6, we generated mice deficient in HDAC6 and found that these animals are viable and display no detectable phenotype, which is consistent with previous observations (Zhang et al, 2008). Moreover, brain morphology was normal in *Hdac6*^*−/−*^ mice. This supports previous findings from *Drosophila* models where knockdown of *Hdac6* did not cause any detectable phenotype in eye morphology, a commonly used system to assess neurodegeneration in invertebrates (Pandey et al, 2007). Notably, we did not detect a compensatory regulation of other HDACs. Specifically, the levels of SIRT2, another known regulator of tubulin acetylation (Tang & Chua, 2008), were similar in *Hdac6*^*−/−*^ mice and wild type littermates. These data suggest that HDAC6 is dispensable for normal development in mice. Moreover, we could not detect changes in the levels of hippocampal bulk histone acetylation or the expression of learning-regulated genes in *Hdac6*^*−/−*^ mice compared to wild type littermates. These data are in line with the view that unlike other HDACs, HDAC6 does not directly affect chromatin plasticity (Fischer et al, 2010; Valenzuela-Fernández et al, 2008). Concordant with the finding that tubulin is a major substrate of HDAC6 (Haggarty et al, 2003; Hubbert et al, 2002; Zhang et al, 2008), α-tubulin K40 acetylation was elevated in the cortex and hippocampus of *Hdac6*^*−/−*^ mice. Moreover, in agreement with the finding that brain morphology was normal in *Hdac6*^*−/−*^ mice, we observed that exploratory behaviour, basal anxiety, motor coordination and long-term associative memory consolidation were indistinguishable between *Hdac6*^*−/−*^ mice and wild type littermates. Spatial memory formation in the water maze paradigm was even slightly improved in *Hdac6*^*−/−*^ mice. The fact that loss of *Hdac6* has almost no effect on cognition is interesting. So far, only class I HDACs have been analysed for their role in memory function using knockout mice and previous studies have shown that loss of *Hdac2* (Guan et al, 2009) and *Hdac3* (McQuown et al, 2010) could enhance memory function and synaptic plasticity in mice. These findings support the view that targeting class I HDACs enhances memory function (Kilgore et al, 2010). However, the role of other classes of HDACs in memory function still remains to be elucidated.

While loss of *Hdac6* did not severely affect cognitive function under basal conditions, we found that spatial and associative memory functions were restored in a mouse model for AD when *Hdac6* was knocked out. To this end, APPPS1-21 mice that lacked *Hdac6* showed enhanced associative and spatial memory functions when compared to APPPS1-21 that express *Hdac6*. Aβ plaque load was not affected in APPPS1-21 and APPPS1-21 *Hdac6*^*−/−*^ mice suggesting that the improvement of cognition was not due to reduced Aβ load. This view is supported by previous studies where therapeutic effects on cognition have been observed in models for AD despite unaltered Aβ plaque load (Govindarajan et al, 2011; Ricobaraza et al, 2009). Especially, since global loss of *Hdac6* does not cause any detectable detrimental phenotype, our data clearly suggest that targeting HDAC6 could be a beneficial therapeutic strategy to treat AD. While our data provides genetic evidence that reducing endogenous HDAC6 levels protects against memory impairment in an AD mouse model, it remains to be tested whether pharmacological inhibition of HDAC6 would have similar effects. In line with our data, recent studies have shown that inhibition of HDAC6 in cortical neurons could rescue neurotoxicity linked to oxidative stress (Rivieccio et al, 2009). Another recent study found that inhibition of HDAC6 rescues axonal degeneration in a model of Charcot-Marie-Tooth disease (d'Ydewalle et al, 2011). However, the therapeutic effect of targeting HDAC6 may not readily translate to all conditions of cognitive impairment and other neurodegenerative diseases. For example, we found that loss of *Hdac6* did not rescue age-related memory disturbances (Supporting Information Fig S2). Moreover, in a *Drosophila* model for the poly-Q disease Spinal Bulbar Muscle Atrophy, in which the ubiquitin proteasome system was inhibited, HDAC6 was found to mediate a compensatory increase in autophagy that was linked to neuroprotection (Pandey et al, 2007). Inhibition of HDAC6 has been shown to be protective in Huntington's disease (HD). In cortical neurons, mutated huntingtin protein impairs intracellular transport of neurotrophic factors such as BDNF. Interestingly, reducing HDAC6 activity was able to rescue this phenotype, which was linked to increased tubulin acetylation (Dompierre et al, 2007). However, while loss of *Hdac6* in a mouse model for HD resulted in increased microtubule acetylation, it failed to rescue neurodegenerative phenotypes and deficits in motor coordination (Bobrowska et al, 2011). While the precise role of HDAC6 in poly-Q diseases needs to be studied in greater detail, a direct role for HDAC6 in the pathogenesis of AD is suggested by the finding that HDAC6 protein levels are increased in postmortem tissues samples from AD patients (Ding et al, 2008). Consistently, postmortem analysis revealed reduced α-tubulin K40 acetylation in NFT-containing neurons from AD patients (Hempen & Brion, 1996). Similar effects were detected in neurons upon treatment with Aβ peptides (Henriques et al, 2010). In line with these findings, we observed increased HDAC6 levels in APPPS1-21 mice. Consequently, the levels of tubulin K40ac were decreased in APPPS1-21 mice, a phenotype that was rescued in these mice upon loss of *Hdac6*. This finding indicates that the therapeutic effect of HDAC6 inhibition in APPPS1-21 mice could be linked to altered tubulin acetylation, which is known to regulate microtubule dynamics and intracellular transport (Creppe et al, 2009; Reed et al, 2006). This view is supported by data indicating that Aβ peptides lead to cytoskeletal abnormalities, specifically the impairment of intracellular transport (Henriques et al, 2010; Stokin et al, 2005). Mitochondrial trafficking, which is essential for normal neuronal function and integrity, was found to be impaired in neurons treated with Aβ peptides (Rui et al, 2006). This corroborates findings from postmortem studies showing abnormal intraneuronal mitochondrial distribution in the brains of AD patients (Wang et al, 2009). It is therefore interesting to note that HDAC6 activity has previously been implicated in intracellular transport (Deribe et al, 2009), including mitochondrial trafficking (Chen et al, 2010). In line with these data, we found that administration of ADDLs to hippocampal neurons isolated from wild type mice caused an impairment of mitochondrial trafficking. In contrast, neurons derived from *Hdac6*^*−/−*^ mice did not show any significant impairment in mitochondrial trafficking upon ADDL treatment. Furthermore, we observed a greater accumulation of mitochondria in neuronal somata compared to the stratum radiatum in the hippocampi of APPPS1-21 mice, but not APPPS1-21-*Hdac6*^*−/−*^ mice, compared to wild type controls. These results suggest that loss of *Hdac6* is protective against Aβ-induced impairment of mitochondrial trafficking in hippocampal neurons *in vitro* as well as *in vivo*. Therefore, increased tubulin acetylation linked to mitochondrial trafficking might provide one, however most likely not the only, cellular substrate for improved cognitive function observed in APPPS1-21 mice that lack *Hdac6*.

In conclusion, our study shows that reducing endogenous HDAC6 levels ameliorates memory impairment in a mouse model for neurodegeneration. These data support the view that the neuroprotective and neuroregenerative effects of pan-HDAC inhibitors are not exclusively due to chromatin remodelling but may also involve modifications of non-histone proteins such as α-tubulin. Especially, the fact that complete loss of *Hdac6* did not cause any overt phenotype but improved cognitive function in a disease model strongly suggests that targeting HDAC6 could be a promising strategy to treat cognitive impairment in neurodegenerative diseases such as AD.

## MATERIALS AND METHODS

### Cell culture

Primary hippocampal cultures were prepared from newborn mouse pups (P0). Briefly, pups were sacrificed by decapitation and hippocampi were isolated after incising the skull and meninges. Papain digestion and mechanical trituration were performed to yield a homogenous cell suspension. Cells were counted using the Neubauer chamber and subsequently plated on 13 mm glass cover slips, pre-coated with 1% w/v poly-d-lysine, in 24-well plates for immunocytochemistry and pre-coated 35 mm glass bottom culture dishes (MatTek Corporation, Ashland, MA, USA) for live imaging. Cultures were incubated at 37°C, 5% CO_2_ and 60% relative humidity and treated with floxuridine 4 days after plating to restrict astrocytic growth. The cultures were nourished by half-medium changes after 7 DIV. Experiments were performed at the age of 15 DIV.

### Aβ oligomerization and treatment

Lyophilized monomeric Aβ1-42 was purchased from Innovagen, Lund, Sweden and stored at −20°C. Preparation of ADDLs was performed as published previously (Klein, 2002). Cultures were treated with 500 nM ADDL solution for 30 or 60 min.

### Live cell imaging

Primary hippocampal cultures grown in 35 mm glass bottom cell culture dishes (MatTek Corporation, Ashland, MA, USA) were treated with 10 mM MitoTracker Red FM dye (Life Technologies, Darmstadt, Germany) for about 30 min to label all mitochondria. Mitochondrial trafficking was analysed by spinning disk confocal microscopy (PerkinElmer, Rodgau, Germany). Cultures were maintained at 37°C and 5% CO_2_ in a humid incubator mounted on the microscope stage. Live imaging was performed by taking an image every 30 s for 5 min prior to and 30 or 60 min after ADDL treatment. The videos were analysed using the MetaMorph® software (Olympus, Germany).

### Animals

Mice were housed under standard housing conditions with food and water *ad libitum* and experimental procedures were approved by the local Animal Care Committee. *Hdac6* knockout mice were generated by replacing exons 10–13 of the *Hdac6* gene with a neomycin resistance gene. Unless otherwise stated all experiments were performed in male mice. To study the loss of *Hdac6* in an AD mouse model, the *Hdac6* knockout mice were crossed with double transgenic APPPS1-21 mice (Radde et al, 2006) and cognition was analysed at the age of 8 months followed by molecular analysis.

### Behavioral analysis

Behavior tests were performed as described before (Govindarajan et al, 2011; Kuczera et al, 2010). In brief, exploratory behaviour was tested using the Open Field. Mice were exposed to a square open arena (80 cm) with an opaque base and transparent walls (20 cm high). Basal anxiety was assessed using the Elevated Plus-Maze situated at a height of 53 cm from the ground. Each rectangular arm measured 45 × 10 cm^2^ with two opposite arms closed on three sides with 30 cm tall opaque walls. The central open field measured 10 × 10 cm^2^. In both tests, the area was evenly lit using dim incandescent bulbs. Each mouse was allowed to explore the area for 5 min and its activity was recorded using the VIDEOMot2 (version 5.72) video tracking system (TSE, Berlin, Germany). The surface was cleaned with 70% ethanol after testing each mouse. For the elevated plus maze test mice were placed in the centre region of the elevated maze facing the open arm. The behaviour was recorded for 5 min using the VideoMot2 system (TSE, Berlin, Germany). To evaluate associative memory, we employed the Fear Conditioning paradigm (TSE, Berlin, Germany). The training consisted of a single exposure to a novel context for 3 min, followed by a single electric foot shock (0.7 mA constant current for 2 sec). Contextual associated memory was analysed 24 h later via assessment of freezing behaviour. Spatial learning was assessed using the Morris Water Maze task in a circular pool of opaque water with a submerged platform. Visual cues were provided for spatial orientation. Each mouse was subjected to four trials each 1 min long for several days. The mice were placed on the platform for 15 s after every trial. Training was discontinued when one of the groups succeeded in locating the platform within 10 s. After 24 h, a probe test was carried out by exposing the mice to the pool for 1 min without the platform. The mouse was tracked using the VIDEOMot2 (version 5.72) video tracking system (TSE, Berlin, Germany). Motor function was assessed on the Rotarod test (TSE, Berlin, Germany). Each mouse was subjected to four habituation sessions (10 rpm constant) and four testing sessions (5–40 rpm uniform acceleration for 3 min, 40 rpm constant for 1 min) on the rotating rod. The performance was assessed by measuring the time spent on the rod.

### Brain extraction and storage

For molecular analysis, mice were sacrificed by cervical dislocation and specific brain regions were isolated by manual dissection, frozen in liquid N_2_ in 1.5 ml tubes and stored at −80°C. For histological analysis, brains were fixed by immersion in 4% PFA for 24 h at 4°C followed by dehydration in a 30% sucrose solution (in 0.01 M PBS) and then frozen over liquid N_2_ and stored at −80°C until further use.

### RNA and protein isolation

Total RNA and proteins were isolated using the TRIzol reagent (Life Technologies, Darmstadt, Germany) according to the manufacturer's instructions. The final RNA pellet was dissolved in 30 µl RNase free ddH_2_O and proteins were dissolved in 3 M urea in 0.01 M PBS using an ultrasonic homogenizer for 10 s, at 8 cycles and 85% power (Bioruptor, Diagenode, Liège, Belgium).

**The paper explained**

PROBLEM:

Alzheimer's disease (AD) is a debilitating neurological disorder that leads to severe loss of memory and cognitive ability in the elderly resulting in a drastic decline in quality of life. Learning and memory are highly specialized brain functions that are executed by neurons in distinct brain regions such as the hippocampus and are essential to our survival. Impaired functioning of neurons is known to underlie the decline in learning and memory in AD patients. Neuronal dysfunction in AD involves the dysregulation of many molecular processes such as gene expression, protein modification and folding, and intracellular transport. Therefore, to treat cognitive pathology in AD effectively, we need to understand the mechanisms that regulate these neuronal processes. The currently available forms of AD therapy have not been successful in restoring neuronal function and relieving these cognitive symptoms in AD.

RESULTS:

We have studied the role of the protein histone deacetylase 6 (HDAC6) in cognition and Alzheimer's disease. HDAC6 catalyses the removal of an acetyl group from acetylated proteins such as α-tubulin. We have discovered that reducing endogenous HDAC6 protein levels in the brains of mice that exhibit certain pathological features of AD improved memory function in these mice. At the molecular level, reducing endogenous HDAC6 levels was found to protect neurons against amyloid-β-induced impairment of mitochondrial trafficking.

IMPACT:

Out study shows that HDAC6 could be a novel therapeutic target to treat AD. Reducing HDAC6 levels did not impair brain functions in healthy mice but improved cognition in a mouse model for AD. This suggests that targeting HDAC6 could be beneficial in ameliorating cognitive pathology in AD patients.

### Subcellular fractionation

Total cellular proteins were separated into nuclear, cytosolic, membrane and cytoskeletal fractions using the ProteoExtract® Subcellular Proteome Extraction Kit (EMD Chemicals Group, Merck KGaA, Darmstadt, Germany) according to the manufacturer's instructions.

### Quantitative real-time PCR (qPCR)

qPCR was performed using a Roche 480 Light Cycler (Roche, Mannheim, Germany). cDNA was synthesized from 1 µg of total RNA using the iScript cDNA Synthesis Kit (BIO-RAD, Hercules, USA) according to the manufacturer's instructions. Expression of individual genes was analysed using the Roche Universal Probe Library (UPL). The housekeeping gene hypoxanthine phosphoribosyltransferase 1 (*Hprt1*) was used as an internal reference for gene expression analysis. The primers and UPL probes used are described in Table 1.

**Table 1:** Primers and UPL probes used for qPCR

Primer	Sequence (5′–3′)	UPL probe
*Acly* F	GCCCTGGAAGTGGAGAAGAT	#10
*Acly* R	CCGTCCACATTCAGGATAAGA	#10
*Fmn2* F	AACAGCAGAAGCCTTTTGTCA	#89
*Fmn2* R	TTCTGCCAGTGGGAAGACA	#89
*GluR1* F	GCCCAATGCAGAGCTCAC	#100
*GluR1* R	GTCACTCCACTCGAGGTAAC	#100
*Gsk3a* F	GAGCCACAGATTACACCTCGT	#76
*Gsk3a* R	CTGGCCGAGAAGTAGCTCAG	#76
*Hprt1* F	TCCTCCTCAGACCGCTTTT	#95
*Hprt1* R	CCTGGTTCATCATCGCTAATC	#95
*Igf2* F	CGCTTCAGTTTGTCTGTTCG	#40
*Igf2* R	GCAGCACTCTTCCACGATG	#40
*Igfbp7* F	CCCTCCATGAAATACCACTGA	#110
*Igfbp7* R	GGCTGTCTGAGAGCACCTTT	#110
*Marcksl1* F	GGCAGCCAGAGCTCTAAGG	#19
*Marcksl1* R	TCACGTGGCCATTCTCCT	#19
*Myst4* F	GCAACAAAGGGCAGCAAG	#19
*Myst4* R	AGACATCTTTAGGAAACCAAGACC	#19
*Ncdn* F	GCTCCTTAGCACCTCTCCAG	#75
*Ncdn* R	GCAGCTGCGAAGAAACCT	#75
*Prkca* F	ACAGACTTCAACTTCCTCATGGT	#60
*Prkca* R	CTGTCAGCAAGCATCACCTT	#60
*Shank3* F	AGGACGTCCGCAATTACAAC	#97
*Shank3* R	AAGCTCAAAGTTCCCTGCAA	#97
*Snap25* F	GCTCCTCCACTCTTGCTACC	#88
*Snap25* R	CAGCAAGTCAGTGGTGCTTC	#88
*Hdac1* F	TGCTGGACTTACGAAACAGC	#81
*Hdac1* R	GTCGTTGTAGGGCAGCTCAT	#81
*Hdac2* F	CTCCACGGGTGGTTCACT	#45
*Hdac2* R	CCCAATTGACAGCCATATCA	#45
*Hdac3* F	TTCAACGTGGGTGATGACTG	#32
*Hdac3* R	TTAGCTGTGTTGCTCCTTGC	#32
*Hdac4* F	CACACCTCTTGGAGGGTACAA	#53
*Hdac4* R	AGCCCATCAGCTGTTTTGTC	#53
*Hdac5* F	GAGTCCAGTGCTGGTTACAAAA	#105
*Hdac5* R	TACACCTGGAGGGGCTGTAA	#105
*Hdac6* F	GAAGGAGGAGCTGATGTTGG	#64
*Hdac6* R	TCATGTACTGGGTTGTCTCCAT	#64
*Hdac7* F	GCCCTTGAGAGAACAGTCCA	#45
*Hdac7* R	CCAAGGGCTCAAGAGTTCTG	#45
*Hdac9* F	TTGCACACAGATGGAGTGG	#32
*Hdac9* R	GGCCCATAGGAACCTCTGAT	#32
*Hdac10* F	TTCCAGGATGAGGATCTTGC	#60
*Hdac10* R	ACATCCAATGTTGCTGCTGT	#60
*Hdac11* F	ATCATGGCAGGGAAGCTG	#77
*Hdac11* R	CACTGGAGCAGTGGTGGA	#77
*Sirt1* F	TCGTGGAGACATTTTTAATCAGG	#104
*Sirt1* R	GCTTCATGATGGCAAGTGG	#104
*Sirt2* F	CACTACTTCATCCGCCTGCT	#66
*Sirt2* R	CCAGCGTGTCTATGTTCTGC	#66
*Sirt3* F	TGCTACTCATTCTTGGGACCTC	#7
*Sirt3* R	GGGCACTGATTTCTGTACTGC	#7
*Sirt5* F	CCAGCTTTAGCAGGAAAAGG	#21
*Sirt5* R	GACTGGGATTCTGGCGTCT	#21
*Sirt6* F	ACGCGGATAAGGGCAAGT	#82
*Sirt6* R	CTCCCACACCTTGCGTTC	#82
*Sirt7* F	TGCAACTCCTCATGAATGAACT	#80
*Sirt7* R	CGCCAAGGAGAAGATTGG	#80
*Hprt1* F	TCCTCCTCAGACCGCTTTT	#95
*Hprt1* R	CCTGGTTCATCATCGCTAATC	#95

### Immunoblot

Proteins were separated via SDS–PAGE and transferred to a nitrocellulose membrane at 4°C overnight (Mini-PROTEAN Tetra Electrophoresis System, BIO-RAD, Munich, Germany). The membrane was rinsed briefly in 0.01 M PBS at room temperature (RT) and incubated in 5% milk prepared in 0.01 M PBS at RT for 1 h to block non-specific sites. Primary antibody dissolved in 0.5% milk in 0.01 M PBS was used to probe the membrane at 4°C overnight on an orbital shaker. Antibodies used were: anti-acetyl-H4K12 (1:5000, Abcam), anti-acetyl-H4K5 (1:2000, Millipore), anti-acetyl-H4K8 (1:1000, Millipore) and anti-acetyl-H4K16 (1:2000, Millipore), anti-acetyl-H3K9 (1:5000, Millipore), anti-acetyl-H3K14 (1:2500, Millipore), anti-HDAC6 (1:1000, Upstate), anti-acetyl-α-tubulin (1:1000, Sigma–Aldrich), anti-SYP (1:1000), and anti-β-actin (1:1000, Santa Cruz). IRDye 800CW- or 680CW-conjugated polyclonal anti-mouse or anti-rabbit IgG cross-adsorbed secondary antibodies (1:15000, LI-COR) were dissolved in 0.5% milk in 0.01 M PBS and added on the membrane for 30 min at RT on a shaker in the dark. Non-specific binding was washed using 0.01 M PBS thrice for 10 min. Detection was performed using an Odyssey IR Scanner (LI-COR, Bad Homburg, Germany).

### Immunohistochemistry

For immunostaining, frozen mouse brains (see above) were sectioned to 30 µm thick sections using a Leica CM 1510S cryostat (Leica Microsystems, Wetzlar, Germany). Sections were stored in a cryoprotectant solution (20% ethylene glycol + 20% glycerol in 0.01 M PBS) in 24-well polystyrene plates at −20°C. Brain sections were washed twice in 0.01 M PBS and incubated in blocking buffer (5% goat serum + 0.3% TritonX-100 in 0.01 M PBS) for 90 min at RT to block non-specific sites and incubated in primary antibody diluted in blocking buffer at 4°C overnight on a shaker. Thereafter, the sections were washed thrice in antibody wash buffer (1% goat serum + 0.2% TritonX-100 in 0.01 M PBS) for 10 min at RT. Secondary antibody incubation was carried out at RT for 2 h with Alexa488- or Cy3-conjugated goat polyclonal anti-rabbit or anti-mouse IgG antibodies dissolved in blocking buffer (1:500, Jackson Immunoresearch Laboratories, Inc., West Grove, PA, USA). The sections were washed thrice in 0.01 M PBS for 10 min, incubated in 4′,6-diamidino-2-phenylindole (DAPI, 10 µg/ml, Sigma–Aldrich, Germany) for 20 min and washed twice in 0.01 M PBS at RT and mounted on SuperFrost glass slides (Menzel-Gläser, Brunswick, Germany). For immunocytochemistry, cells grown on glass cover slips were washed using cold 0.01 M PBS and fixed using 4% paraformaldehyde and immunolabelling was performed as mentioned above in a humid chamber. The following primary antibodies were used: SYP (1:1000, Sigma–Aldrich), NeuN (1:1000, Chemicon), Amyloid-β, 17-24 (4G8, 1:1000, Convance), Tom20 (FL-145, 1:200, Santa Cruz).

### Data analysis

Data were analysed by unpaired student's *t*-test and two-way Analysis of Variance (ANOVA) and *p* < 0.05 was considered significant. Values are displayed as mean ± standard error of mean (SEM).

## Supplementary material

Detailed facts of importance to specialist readers are published as ”Supporting Information”. Such documents are peer-reviewed, but not copy-edited or typeset. They are made available as submitted by the authors.
