# Supplementary material for: Reducing HDAC6 ameliorates cognitive deficits in a mouse model for Alzheimer's disease
Source: EMBO Mol Med. 2012 Nov 26;5(1):52–63. doi: 10.1002/emmm.201201923 (PMC3569653; doi:10.1002/emmm.201201923)
Supplement: Supplementary file 1 [file emmm0005-0052-SD1.pdf]

EMBO Molecular Medicine

Manuscript EMM-2012-01923

# Reducing HDAC6 ameliorates cognitive deficits in a mouse model for Alzheimer's disease

Nambirajan Govindarajan, Pooja Rao, Susanne Burkhardt, Farahnaz Sananbenesi, Oliver M. Schlüter, Frank Bradke, Jianrong Lu, and André Fischer

*Corresponding author: Andre Fischer, German Center for Neurodegenerative Diseases DZNE Goettingen*

---

**Review timeline:**

| | |
|---|---|
| Initial Correspondence: | 13 May 2012 |
| Editorial Decision: | 16 May 2012 |
| Transfer date: | 23 August 2012 |
| Editorial Decision: | 24 September 2012 |
| Revision received: | 04 October 2012 |
| Accepted: | 05 October 2012 |

---

## Transaction Report:

(Note: With the exception of the correction of typographical or spelling errors that could be a source of ambiguity, letters and reports are not edited. The original formatting of letters and referee reports may not be reflected in this compilation.)

## Transfer Note:

Please note that this manuscript was originally submitted to the EMBO Journal where it was peer-review and revised. It was then transferred to EMBO Molecular Medicine with the original referees comments attached. (Please see below)

Original referee comments – The EMBO Journal

---

MS # EMBOJ-20112-81361:

Referee #1 Review:

In this manuscript, Govindarajan et. al. report that the loss of HDAC6 in mice rescues memory impairments in a mouse model for Alzheimer's disease. To obtain a mechanistic understanding, the authors revisited the issue that the hyper-acetylation of tubulin caused by HDAC6 deficiency or inhibition could improve the microtubule-dependent transport of mitochondria, and suggested that this activity may contribute to its therapeutic effect. In principle, this report could have an important therapeutic implication for Alzheimer's disease. However, the author's conclusion is premature and needs additional experimental support.
Two assays were used to probe learning and memory functions. While it is interesting that the behavioral defect in APPS1-21 is largely rescued by HDAC6 deficiency in both assays, additional memory assays would be required to reach the key conclusion that "these data suggest that that loss of Hdac6 can restore memory function in a mouse model for AD". Since it is formally possible that

the effect of HDAC6 deficiency could be developmental, a phenotypic recusal by a HDAC6-selective inhibitor would also greatly enhance the significance of this report.
The authors suggested that the hyper-acetylation of tubulin contributes to mitochondrial transport efficiency. Although the HDAC6 specific inhibitor has previously shown to expedite mitochondrial transport (PLoS One. 2010 May 26;5(5):e10848.), whether acetylation of tubulin itself is responsible for the phenotype is not known. The application of KR (mimic deacetylated form) and KQ (mimic acetylated form) mutants of tubulin might strengthen the mechanistic foundation of this report. Further, there is no clear evidence that mitochondrial transport plays a critical role in the loss of memory function or cell death associated with APP. Without additional evidence supporting this proposition, the link of HDAC6 to mitochondrial transport, in itself, is no longer novel.
In summary, while this study has uncovered an interesting role of HDAC6 on APP-induced memory deficiency, additional mechanistic study or a pharmacological rescue experiment would be needed for its publication at the EMBO Journal (see specific comment).

Additional comments,

1. In Figure 2A, 2B, 2C, the authors concluded that HDAC6 deficient mice is normal in basal anxiety, which contradicts to a recent study based on the same HDAC6 KO strain (PLoS One. 2012;7(2):e30924). Also, result in Figure 2A does not seem to match that in Figure 2B and 2C, since it looks like that HDAC6 KO mice stay less at periphery and travel less.
2. In Figure 2H, the authors showed that spatial memory is slightly increased in HDAC6 KO mice but mitochondrial transport is not increased in primary neuron from HDAC6 KO mice without ADDL treatment (Figure 4H). It suggests that mitochondrial transport may be less important to improve spatial memory function. It needs to be discussed.
3. In fear conditioning experiment, sensitivity to foot-shock should be run as a control for the wild-type, APPPS1-21, and APPPS1-21-HDAC6-/- mice.
4. In figure 4H, the authors showed that the loss of HDAC6 could blunt the impairment of mitochondrial transport by ADDL treatment. However, it is more critical to demonstrate the impairment of mitochondrial transport in primary neuron from APPPS1-21-HDAC6 -/- mice comparing those from APPPS1-21 mice, since extracellular ADDL treatment is different from intracellular expression of APPPS1-21.
5. Since HDAC6 gene is located in X chromosome, were female knockout mice used in the study? The genotype was denoted HDAC6-/-. The authors should include the information about sex and age of mice used for behavior experiments.
6. The authors have previously reported that sodium butyrate can improve memory function in the APPPS1-21 model. Since sodium butyrate does not inhibit HDAC6 activity on tubulin acetylation, the authors should discuss on these two findings.
7. Background information on the APPPS1-21 model should be provided.
8. Literature citation in this article is not very precise. Both primary literature and review were cited at the same time. For clarity and proper credits, the authors should mark review article as "reviewed in XXXX".

Referee #2 Review:

The authors report that HDAC6 deficiency prevents memory impairment and tubulin acetylation in a mouse model for Alzheimer's disease. They provide some evidence that this beneficial effect of HDAC6 deficiency is associated with maintenance of microtubule dynamics and mitochondrial transport. The authors conclude that: "Thus, our study indicates that targeting HDAC6 could be a suitable strategy to ameliorate cognitive decline observed in Alzheimer's disease." While interesting, there are several major concerns that reduce confidence in the authors' conclusions.

1. The authors claim that HDAC6 deficiency "rescues" cognitive deficits in APP/PS1 transgenic mice. This is not correct. In order to "rescue" a deficit, the deficit must first exist. In this case, there is a "prevention" or more likely a "delay in the onset" of the cognitive deficit.

2. Because HDAC6 is absent from the beginning of development in the HDAC6 KO mice, it is important that a rescue experiment is performed in which HDAC6 expression is re-instated in neurons in the brains of adult HDAC6-/- APP/PS1 mice. This can be accomplished using a viral

vector (adenovirus or lentivirus) to express HDAC6.

3. The possibility that HDAC6 inhibition may be a strategy for ameliorating cognitive decline in AD is a speculative stretch, based upon the small amount of data in the present study. Are there selective inhibitors of HDAC6 that could be used in a preclinical study to support the potential of drugs that inhibit this enzyme as a treatment for AD?

Transfer – author comments – initial correspondence 13 May 2012

The editor of The EMBO Journal told me that she had contacted you regarding a manuscript we had submitted to EMBO J that might be more suitable for EMBO Molecular Medicine. I would appreciate the chance to discuss this option with you and send you some additional background info along.

The manuscript is currently titled "Targeting HDAC6 rescues cognitive deficits in a mouse model for Alzheimer's disease" and is to our knowledge the first study in which an HDAC knockout mouse is crossed to a mouse model for Alzheimer's disease (AD). The study was initiated on the basis that recent research suggests that administration of histone deacetylase (HDAC) inhibitors can ameliorate cognitive deficits in AD animal models. The translation of these promising findings into novel drugs is so far hindered by the fact that on one hand the employed pan-HDAC inhibitors have limited specificity to distinct HDAC proteins while on the other little is known about the role of individual HDAC proteins in the adult brain. Moreover, it is important to note that although the therapeutic effect of HDAC inhibition has been mainly discussed in the context of histone acetylation and gene expression, HDACs are known to target other proteins than histones in all subcellular compartments. Thus it is unclear to what extent the therapeutic effect of targeting HDACs is due to the reinstatement of genome plasticity or other additional mechanisms.

In the present study we started to address these questions by studying the role of HDAC6 in memory function and AD pathogenesis. HDAC6 is a unique member of the HDAC family in that it acts mainly on cytoplasmic non-histone substrates. We generated Hdac6 knockout mice (Hdac6 -/- mice) and show that loss of HDAC6 increases hippocampal a-tubulin lysine 40 acetylation but has no effect on histone acetylation. Hdac6 -/- mice are viable and show no detectable cognitive dysfunction. However, when crossed with a model for severe amyloid pathology, loss of HDAC6 rescued a-tubulin lysine 40 acetylation and restored memory function. On the mechanistic level, we identify one cellular substrate to explain, at least in part, the therapeutic effect of HDAC6 deficiency by showing that loss of Hdac6 rescues Ab-induced deficits in mitochondrial trafficking in neurons. In conclusion, our data suggest that strategies to manipulate HDAC6 activity could open a suitable novel therapeutic avenue to treat AD. Our data is of utmost importance to the scientific community and provides important information in a very rapidly advancing field of research. Therefore, publishing these results at the earliest would greatly benefit a large community of researchers and kindle the development of further exciting projects along these lines. We are confident that our manuscript is of interest to the broad readership of EMBO Molecular Medicine and will be cited as a key publication in the field.

The study has been reviewed by two referees for EMBO J. Both referees acknowledge the importance our findings but ask for a number of additional experiments that in our opinion are much beyond the scope of our study and would not add to the key message that we aim to communicate.

Please find below our response to the specific comments made by the referees.

REFEREE #1

Referee 1 has three major remarks:
Referee #1 says 'Two assays were used to probe learning and memory functions. While it is interesting that the behavioral defect in APPS1-21 is largely rescued by HDAC6 deficiency in both

assays, additional memory assays would be required to reach the key conclusion that "these data suggest that that loss of Hdac6 can restore memory function in a mouse model for AD".'

We appreciate this comment. We have tested two types of memories, associative and spatial memory consolidation, using two well-established tests that are relevant in the context of Alzheimer's disease pathology. We also strengthen our results by testing the mice for motor coordination (rotarod) and explorative behavior (open field test). We do not think that adding another memory test would significantly strengthen our conclusion. We also cannot think of a suitable test that would add significant novel information. Moreover, to obtain the necessary number of animals at the appropriate age to perform another memory test or repeat the water maze test for example in a modified version would only significantly delay publication of our results in a very competitive area of research. In specific response to this comment we suggest to change our wording and specifically state that loss of Hdac6 specifically affects associative and spatial memory formation rather that stating that 'memory function' is restored to avoid any possible misinterpretation of our findings.

Referee # 1 states 'Since it is formally possible that the effect of HDAC6 deficiency could be developmental, a phenotypic recusal by a HDAC6-selective inhibitor would also greatly enhance the significance of this report.'
This is a valid point. However, the aim of our study was to elucidate the role of HDAC6 in Alzheimer's disease since the current data does not allow for a clear conclusion and the field desperately needs clear data on which one of the 11 HDACs would be the most suitable drug target. The currently available HDAC inhibitors act as pan-inhibitors (see our introduction) making it difficult to assess their effectiveness. Thus using Hdac6 ko mice provides, in our opinion, the most conclusive evidence. In fact, our study is the first in which an Hdac ko mouse is crossed with a mouse model for Alzheimer's disease. While most HDAC inhibitors affect multiple HDACs, it is true that compounds such as tubastatin appear to be more selective for HDAC6 than for other HDACs. However, as with all pharmacological approaches, specificity depends to the dose, bioavailability, etc. In collaboration with a chemical epigenetics group we are currently trying to develop HDAC6-specific inhibitors that cross the Blood brain barrier and subsequently plan to find a dose in vivo that would affect ñ ideally ñ only HDAC6. However, these data cannot be provided on a reasonable time scale on which we feel the data presented here should be made available to the scientific community.

Referee # 1 furthermore comments 'The authors suggested that the hyper-acetylation of tubulin contributes to mitochondrial transport efficiency. Although the HDAC6 specific inhibitor has previously shown to expedite mitochondrial transport (PLoS One. 2010 May 26;5(5):e10848.), whether acetylation of tubulin itself is responsible for the phenotype is not known. The application of KR (mimic deacetylated form) and KQ (mimic acetylated form) mutants of tubulin might strengthen the mechanistic foundation of this report. Further, there is no clear evidence that mitochondrial transport plays a critical role in the loss of memory function or cell death associated with APP. Without additional evidence supporting this proposition, the link of HDAC6 to mitochondrial transport, in itself, is no longer novel.'

We agree that to understand in detail how tubulin acetylation affects mitochondrial transport in neurons is a highly interesting point. However, we feel this comment is unfair for two reasons. (1) To provide such data would be a project by itself and would moreover only dilute our message. (2) To our knowledge there is one study that exclusively addressed the role of tubulin acetylation in cellular transport (Reed et al., Curr Biol., 2006) and one study that addressed specifically the role of tubulin tyrosination in cellular transport (Konishi et al., Nature Neurosci, 2009). To provide now the mechanisms by which in neurons tubulin acetylation affects cellular transport, for example of mitochondria, is beyond the scope of our study. We have initiated a collaboration in which a newly recruited PhD student is addressing this issue and we hope to have results ready for publication within the next 3 years.
We strongly disagree with the comment that our data are not new. This is not true. We decided to analyze mitochondrial trafficking because there is recently published evidence that A  affects this process and that in turn HDAC6 regulates mitochondrial transport. We describe this in detail in our manuscript and cite these studies. Nevertheless, it has never been shown that A -induced impairment of mitochondrial trafficking is rescued in Hdac6-deficient neurons.

Referee #1 has some additional minor remarks that can easily be addressed.

© EMBO

REFEREE #2

He or she has 3 comments.

Referee #2 says 'The authors claim that HDAC6 deficiency "rescues" cognitive deficits in APP/PS1 transgenic mice. This is not correct. In order to "rescue" a deficit, the deficit must first exist. In this case, there is a "prevention" or more likely a "delay in the onset" of the cognitive deficit.'

We would be happy to rephrase this statement.

Referee #2 says 'Because HDAC6 is absent from the beginning of development in the HDAC6 KO mice, it is important that a rescue experiment is performed in which HDAC6 expression is re-instated in neurons in the brains of adult HDAC6-/- APP/PS1 mice. This can be accomplished using a viral vector (adenovirus or lentivirus) to express HDAC6.'

If interpreted correctly, the hypothesis would be that the presence of HDAC6 in APPPS1-21 mice would allow the development of disease phenotypes. Besides the fact that also APP/PS1 is expressed from early states of development we would argue that the employed APP HDAC6 +/+ mice are the perfect control to address this concern. We assume that this comment is not very precisely articulated and infact goes into the same direction as comment 2 made by referee 1. Thus, please see response to comment 2, referee 1.

This referee also says 'The possibility that HDAC6 inhibition may be a strategy for ameliorating cognitive decline in AD is a speculative stretch, based upon the small amount of data in the present study. Are there selective inhibitors of HDAC6 that could be used in a preclinical study to support the potential of drugs that inhibit this enzyme as a treatment for AD?'

Please see response to comment 2 by referee 1.

1st Editorial Decision 16 May 2012

Thank you for your letter and the point-by-point response you provided. I now had the time to consider both as well as to read the manuscript, reviewer reports and related literature again. I have also discussed them with the editorial team. We agree that the study is potentially interesting but also that additional experimental evidence for the proposed mechanism is needed for consideration at EMBO Molecular Medicine.

We agree that addressing the concerns regarding developmental effects of HDAC KO raised by both reviewers as well as the concern raised by Reviewer #1 regarding tubulin hyper-acetylation and its link to mitochondrial transport is beyond the scope of the study. However, we feel that the second points raised by this reviewer in the same paragraph ("Further, there is no clear evidence that mitochondrial transport plays a critical role in the loss of memory function or cell death associated with APP.") is crucial. It remains open whether the affected mice display abnormal mitochondrial transport and whether this potential defect is indeed linked to the memory defects or Abeta toxicity. If you are willing and able to experimentally address these points, we would be glad to consider an extended version of the paper. Otherwise, I am sorry that I could not bring better news.

Yours sincerely,

Editor

EMBO Molecular Medicine

Transfer 23 August 2012

We had previously communicated regarding our manuscript initially submitted to EMBO J and which was then found to be more suitable for EMBO Molecular Medicine.

The manuscript is now entitled "***Reducing HDAC6 ameliorates cognitive deficits in a mouse model for Alzheimer's disease***" and is to our knowledge the first study in which a HDAC knock out mouse is crossed to a mouse model for Alzheimer's disease.

The study was initiated on the basis that recent research suggests that administration of histone-deacetylase (HDAC) inhibitors can ameliorate cognitive deficits in AD animal models. The translation of these promising findings into novel drugs is so far hindered by the fact that the employed pan-HDAC inhibitors have limited specificity to distinct HDAC proteins while on the other hand little is know on the role of individual HDAC proteins in the adult brain. Moreover, it is important to note that although the therapeutic effect of HDAC inhibition has been mainly discussed in the context of histone acetylation and gene expression, HDACs are known to target other proteins than histones in all subcellular compartments. Thus it is unclear to what extend the therapeutic effect of targeting HDACs is due to the reinstatement of genome plasticity or other additional mechanisms.

In the present study we started to address these questions by studying the role of HDAC6 in memory function and AD pathogenesis. HDAC6 is a unique member of the HDAC family in that it acts mainly on cytoplasmic non-histone substrates. We generated *Hdac6* knockout mice (*Hdac6* -/- mice) and show that loss of HDAC6 increases hippocampal $\alpha$-tubulin lysine 40 acetylation but has no effect on histone acetylation. *Hdac6* -/- mice are viable and show no detectable cognitive dysfunction. However, when crossed with a model for severe amyloid pathology, reduction of HDAC6 levels ameliorated $\alpha$-tubulin lysine 40 acetylation and memory function. On the mechanistic level, we identify one cellular substrate to explain, at least in part, the therapeutic effect of reducing HDAC6 levels by showing that loss of HDAC6 rescues A$\beta$-induced deficits in mitochondrial trafficking *in vitro* and *in vivo*. In conclusion, our data suggest that strategies to reduce HDAC6 levels could open a suitable novel therapeutic avenue to treat AD.

Our data is of utmost importance to the scientific community and provides important information in a very competitive field of research.

We are confident that our manuscript is of interest to the broad readership of *EMBO Mol. Medicine* and will be cited as a key publication in the field.

With best regards

yours

Andre Fischer

**Additional information of the current version of our manuscript:**

The study has been reviewed by two referees for *EMBO J.* Both referees acknowledged the importance our findings but asked for a number of additional experiments. We have now addressed these concerns in the current manuscript.

Please find below our response to the specific comments made by the referees.

REFEREE #1

Referee 1 has 3 major remarks:

Referee #1 says "*Two assays were used to probe learning and memory functions. While it is interesting that the behavioral defect in APPS1-21 is largely rescued by HDAC6 deficiency in both assays, additional memory assays would be required to reach the key conclusion that "these data suggest that that loss of Hdac6 can restore memory function in a mouse model for AD"*. "

In specific response to this referee we now rewrote our statement and say that reducing endogenous HDAC6 levels ameliorates associative and spatial memory formation rather that stating that "*memory function*" is restored.

We appreciate this comment. We tested two types of memories, associative and spatial memory consolidation, two well-established tests that are relevant in the context of Alzheimer's disease pathology. We also strengthen our results by testing the mice for motor-coordination (rotarod) and explorative behavior (open field test). Adding another memory test would significantly strengthen our conclusion. We also cannot think of a suitable test that would add significant novel information. Moreover, to obtain the necessary number of animals to perform another memory test or repeat the water maze test for example in a modified version would only have the effect to significantly delay publication of our results in a very competitive area of research.

See page 4, line 5 from bottom, page 7 lines 14 from bottom

Referee # 1states that "*Since it is formally possible that the effect of HDAC6 deficiency could be developmental, a phenotypic recusal by a HDAC6-selective inhibitor would also greatly enhance the significance of this report.* "

This is a valid point. However, the aim of our study was to elucidate the role of HDAC6 in Alzheimer's disease since the current data does not allow for a clear conclusion and the field desperately needs clear data which of the 11 HDACs would be the most suitable drug target (see our introduction). Thus using HDAC6 ko mice is in our opinion the most conclusive evidence. In fact our study is the first in which a HDAC ko mouse is crossed with a mouse model for Alzheimer's disease. While most HDAC inhibitors affect multiple HDACs it is true that compounds such as tubastatin appear to be more selective for HDAC6 than for other HDAC6. However, as with all pharmacological approaches specificity depends on the dose, bioavailability etc. In collaboration with a chemical epigenetics group we are currently trying to develop HDAC6 specific inhibitors that cross the Blood brain barrier and subsequently plan to find a dose *in vivo* that would affect – ideally – only HDAC6. However, these data cannot be provided on a reasonable time scale on which we feel the data presented here should be made available to the scientific community.

We specifically acknowledge this point in the current version of our manuscript. See page11 lines 16- 19. Moreover, throughout the manuscript we clearly state that our data suggest that reduction of endogenous HDAC6 ameliorates memory impairments.

Referee # 1 furthermore comments: "*The authors suggested that the hyper-acetylation of tubulin contributes to mitochondrial transport efficiency. Although the HDAC6 specific inhibitor has previously shown to expedite mitochondrial transport (PLoS One. 2010 May 26;5(5):e10848.), whether acetylation of tubulin itself is responsible for the phenotype is not known. The application of KR (mimic deacetylated form) and KQ (mimic acetylated form) mutants of tubulin might strengthen the mechanistic foundation of this report. Further, there is no clear evidence that mitochondrial transport plays a critical role in the loss of memory function or cell death associated with APP. Without additional evidence supporting this proposition, the link of HDAC6 to mitochondrial transport, in itself, is no longer novel.*"

We strongly disagree with the comment that our data is not novel. We decided to analyze mitochondrial trafficking because there is recently published evidence that Aß affects this process and that in turn HDAC6 regulates mitochondrial transport. We describe this in detail in our manuscript and cite these studies. Nevertheless, it has never been shown that Aß-induced impairment of mitochondrial trafficking is ameliorated by reducing HDAC6 levels in neurons.

We now provide novel data to strengthen our arguments. Thus we have now consulted with experts in the field and conducted a detailed analysis of mitochondrial distribution in wild type mice, APPPS-21 and APPPS1-21 HDAC6 -/- mice *in vivo* using Tom 20 immunoreactivity. In line with the data obtained in primary neurons, we show that reduction of HDAC6 levels ameliorates mitochondrial mislocalization in APPPS1-21 mice. The data is now presented as novel figure 4 I and discussed on page 8, last paragraph and page 9.

We agree that to understand in detail how tubulin-acetylation affects mitochondrial transport in neurons is a highly interesting point. We have now consulted on this issue with experts in the field and we feel this comment is not fair. To provide such data is a project by itself and would moreover only dilute our message. To our knowledge there is one study that exclusively addressed the role of tubulin-acetylation in cellular transport (Reed et al., Curr Biol., 2006) and one study that addressed specifically the role of tubulin-tyrosination in cellular transport (Konishi et al., Nature Neurosci, 2009). To provide now the mechanisms by which in neurons tubulin acetylation affects cellular transport, for example of mitochondria, is beyond the scope of our study. We have initiated a collaboration in which a newly recruited PhD student is addressing this issue and we hope to have results ready for publication within the next 3 years.

REFEREE #2

He or she has 3 comments

Referee #2 says "*The authors claim that HDAC6 deficiency "rescues" cognitive deficits in APP/PS1 transgenic mice. This is not correct. In order to "rescue" a deficit, the deficit must first exist. In this case, there is a "prevention" or more likely a "delay in the onset" of the cognitive deficit.* "

We have changes the title of the manuscript accordingly and rewrote corresponding statements throughout the text.

Referee #2 says.: "*Because HDAC6 is absent from the beginning of development in the HDAC6 KO mice, it is important that a rescue experiment is performed in which HDAC6 expression is re-instated in neurons in the brains of adult HDAC6-/- APP/PS1 mice. This can be accomplished using a viral vector (adenovirus or lentivirus) to express HDAC6.*"

If interpreted correctly, the hypothesis would be that the presence of HDAC6 in APP mice would allow the development of disease phenotypes. We would argue that the employed APP HDAC6 +/+ mice are the perfect control to address this concern. We assume that this comment goes into the same direction as comment 2 made by referee 1. Thus, please see response to comment 2, referee 1.

This referee says. "*The possibility that HDAC6 inhibition may be a strategy for ameliorating cognitive decline in AD is a speculative stretch, based upon the small amount of data in the present*

*study. Are there selective inhibitors of HDAC6 that could be used in a preclinical study to support the potential of drugs that inhibit this enzyme as a treatment for AD?* "

Please see response to comment 2 made by referee 1.

2nd Editorial Decision 24 September 2012

Thank you for the submission of your manuscript "Targeting HDAC6 ameliorates cognitive deficits in a mouse model for Alzheimer's disease" to EMBO Molecular Medicine. We have now received the reports from the reviewers who were asked to re-review your manuscript.

As you will see, the Reviewers acknowledge that the manuscript was significantly improved during revision and Reviewer #1 notes that it is suitable for publication. However, Reviewer #2 still raises some concerns that should be convincingly addressed.

On a more editorial note, please address the points raised below:

- Immunoblots should be surrounded by a black line to indicate the borders of the blot, if the background is faint. Please include these in the respective figures.

- The description of all reported data that includes statistical testing must state the name of the statistical test used to generate error bars and P values, the number (n) of independent experiments underlying each data point (not replicate measures of one sample), and the actual P value for each test (not merely 'significant' or 'P < 0.05').

I look forward to reading a new revised version of your manuscript as soon as possible.

Yours sincerely,

Editor
EMBO Molecular Medicine

***** Reviewer's comments *****

Referee #1 (Comments on Novelty/Model System):

A solid study, but of moderate impact.

Referee #1 (Other remarks):

The authors have addressed my concerns adequately.

Referee #2:

The overall conclusion of this report is of potential significance, provide tantalizing data that could lead to important investigation of the effect of HDAC6-selective inhibitors on Alzheimer's disease. It can be accepted for publication after addressing the following comments:
1. The images on new Figure 4I are not very discernible to make out the stated difference in mitochondrial distribution.
2. While it is clear that loss of HDAC6 can suppress the effect of abeta on mitochondrial transport, the significance of this finding remains unclear. At the minimum, the authors should modify the

statement and make it clear that other HDAC6-regulated mechanisms might be involved the partial suppression of the phenotypes observed.

1st Revision - authors' response 04 October 2012

Thank you for your decision letter on our manuscript. Please find enclosed the revised version of our manuscript titled 'Reducing HDAC6 ameliorates cognitive deficits in a mouse model for Alzheimer's disease' to EMBO Molecular Medicine.

We have now made the corrections and changes advised by you and reviewer 2. Below you will find our response to the reviewers comment.

We hope that this version of our manuscript is acceptable for publication in EMBO Molecular Medicine.

Thank you for your consideration.

Response to Reviewers

Referee #1

Referee #1 says: "*The authors have addressed my concerns adequately.*"

We thank this referee for his/her comment.

Referee #2:

*Referee #2 states: "The overall conclusion of this report is of potential significance, provide tantalizing data that could lead to important investigation of the effect of HDAC6-selective inhibitors on Alzheimer's disease. It can be accepted for publication after addressing the following comments:*
*1. The images on new Figure 4I are not very discernible to make out the stated difference in mitochondrial distribution.*
*2. While it is clear that loss of HDAC6 can suppress the effect of abeta on mitochondrial transport, the significance of this finding remains unclear. At the minimum, the authors should modify the statement and make it clear that other HDAC6-regulated mechanisms might be involved the partial suppression of the phenotypes observed."*

We thank this referee for his/her insightful suggestion. In response to the first comment, we have included high-magnification images of Tom20 staining in Fig. 4I to depict the difference in mitochondrial distribution. Please see the rearranged panel I within Fig. 4.

In response to the second comment, we completely agree that elevation of -tubulin acetylation due to loss of Hdac6 may not be the only protective mechanism behind the observed cognitive improvement in the APPPS1-21 mouse model. We clearly state this on page 13, lines 1-4.
