# Supplementary material for: Reducing HDAC6 ameliorates cognitive deficits in a mouse model for Alzheimer's disease
Source: EMBO Mol Med. 2012 Nov 26;5(1):52–63. doi: 10.1002/emmm.201201923 (PMC3569653; doi:10.1002/emmm.201201923)
Supplement: Supplementary file 2 [file emmm0005-0052-SD2.pdf]

Table of Contents

1. Figure S1: Characterization of *Hdac6* -/- mice
2. Figure S2: Associative memory in aged *Hdac6* -/- mice
3. References

Supplemental Material

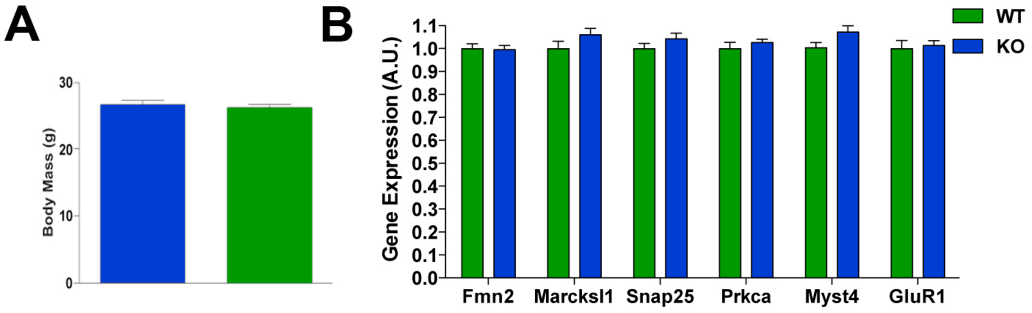

Figure S1: Characterization of *Hdac6* -/- mice

(A) No difference in body mass was detected between 3-5-month-old *Hdac6* -/- (KO) and wild type (WT) mice (n = 10). **(B)**. RNA was isolated from the hippocampi of *Hdac6* -/- and wild type mice and the expression of learning regulated genes[1,2] was analyzed via qPCR. No significant differences were observed (n = 6). Error bars indicate S.E.M.

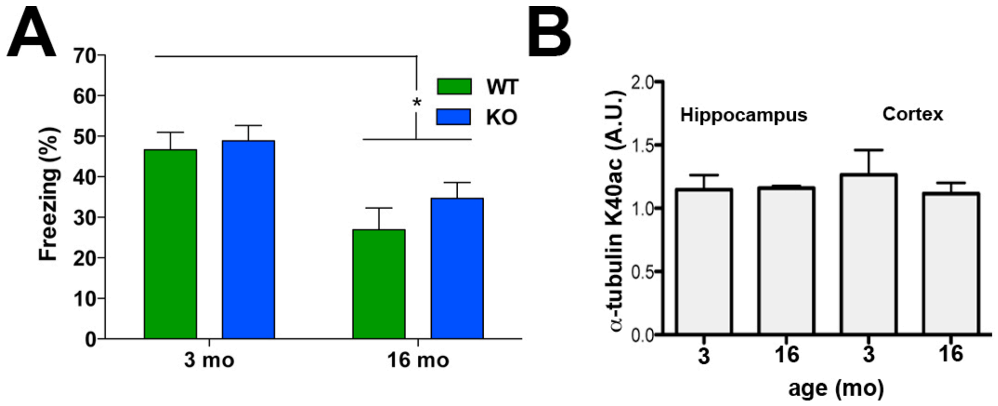

Figure S2: Associative memory in aged *Hdac6* -/- mice

(A) Three and 16-month-old wild type and *Hdac6* -/- mice were subjected to contextual fear conditioning training. Freezing behavior, indicative of associative learning, was analyzed 24 h later. In line with previous data[1], we found that consolidation of associative memory was impaired in 16-month-old wild type when compared to 3-month-old wild type mice ($P < 0.05$). A similar age-dependent impairment was observed in *Hdac6* -/- mice ($P < 0.05$). **(B)** We used quantitative immunoblot analysis to compare hippocampal α-tubulin acetylation at lysine 40 (α-tubulin K40ac) between 3- and 16-month-old wild type mice. No significant difference was observed between groups. In conclusion, these data suggest that HDAC6 is not implicated in age-associated memory disturbances. Error bars indicate S.E.M.

References

1 Peleg, S. *et al.* Altered histone acetylation is associated with age-dependent memory impairment in mice. *Science* **328**, 753-756 (2010).

2 Govindarajan, N., Agis-Balboa, C., Walter, J., Sananbenesi, F. & Fischer, A. Sodium Butyrate Improves Memory Function in an Alzheimer's Disease Mouse Model When Administered at an Advanced Stage of Disease Progression. *Journal of Alzheimer's Disease* **24**, 1-11 (2011).
